# Growth Factors and Their Roles in Multiple Sclerosis Risk

**DOI:** 10.3389/fimmu.2021.768682

**Published:** 2021-10-21

**Authors:** Hui Lu, Peng-Fei Wu, Deng-Lei Ma, Wan Zhang, Meichen Sun

**Affiliations:** ^1^Department of Neurology, Xuanwu Hospital, Capital Medical University, Beijing, China; ^2^Center for Medical Genetics & Hunan Key Laboratory of Medical Genetics, School of Life Sciences, Central South University, Changsha, China; ^3^Department of Neurology, Beth Israel Deaconess Medical Center, Harvard Medical School, Boston, MA, United States; ^4^Department of Pharmacy, Xuanwu Hospital, Capital Medical University, Beijing, China; ^5^Department of Biology, Boston University, Boston, MA, United States

**Keywords:** multiple sclerosis, growth factors, fibroblast growth factor 23, Mendelian randomization, genetic epidemiology

## Abstract

**Background:**

Previous studies have suggested essential roles of growth factors on the risk of Multiple Sclerosis (MS), but it remains undefined whether the effects are causal.

**Objective:**

We applied Mendelian randomization (MR) approaches to disentangle the causal relationship between genetically predicted circulating levels of growth factors and the risk of MS.

**Methods:**

Genetic instrumental variables for fibroblast growth factor (FGF) 23, growth differentiation factor 15 (GDF15), insulin growth factor 1 (IGF1), insulin-like growth factor binding proteins 3 (IGFBP3) and vascular endothelial growth factor (VEGF) were obtained from up-to-date genome-wide association studies (GWAS). Summary-level statistics of MS were obtained from the International Multiple Sclerosis Genetics Consortium, incorporating 14,802 subjects with MS and 26,703 healthy controls of European ancestry. Inverse-variance weighted (IVW) MR was used as the primary method and multiple sensitivity analyses were employed in this study.

**Results:**

Genetically predicted circulating levels of FGF23 were associated with risk of MS. The odds ratio (OR) of IVW was 0.63 (95% confidence interval [CI], 0.49–0.82; *p* < 0.001) per one standard deviation increase in circulating FGF23 levels. Weighted median estimators also suggested FGF23 associated with lower MS risk (OR = 0.67; 95% CI, 0.51-0.87; *p* = 0.003). While MR-Egger approach provided no evidence of horizontal pleiotropy (intercept = -0.003, *p* = 0.95). Results of IVW methods provided no evidence for causal roles of GDF1, IGF1, IGFBP3 and VEGF on MS risks, and additional sensitivity analyses confirmed the robustness of these null findings.

**Conclusion:**

Our results implied a causal relationship between FGF23 and the risk of MS. Further studies are warranted to confirm FGF23 as a genetically valid target for MS.

## Introduction

Multiple sclerosis (MS) is the most common chronic autoimmune disease affecting the central nervous system. The incidence of MS is 2.1 per 100,000 persons/year and approximately 2.8 million people live with MS worldwide (1). It is the leading non-traumatic neurological cause of disability in young individuals (2). The typical pathology of MS is focal areas of demyelination, inflammation and glial reaction in brain, spinal cord and optic nerve (3). The clinical characteristics of MS are intermittent and recurrent episodes of neurological dysfunction, eventually leading to disability and impaired cognition (3). The etiology and mechanisms of MS remain not fully understood. The need for continued studies is compelling to improve our understanding of its nosogenesis.

Growth factors are regulating cytokines in the pathways of cell proliferation, differentiation and activation. Previous studies have suggested growth factors as risk factors for MS and important players in the initiation and progression of MS.

Fibroblast growth factor (FGF) regulates various biological functions, including cellular proliferation, survival, migration and differentiation (4). FGF23 is a critical player in vitamin D metabolism. It is mainly released from osteoblasts. It inhibits 1α-hydroxylase and stimulates 24α-hydroxylase, resulting in the conversion of 25-hydroxyvitamin D into 24,25-dihydroxyvitamin D instead of into 1,25-dihydroxyvitamin D. Growth differentiation factor-15 (GDF15) belongs to the transforming growth factor beta superfamily. It regulates inflammation and apoptosis in various diseases (5–7). Levels of serum GDF15 were positively correlated with the Expanded Disability Status Scale of MS patients (8). Insulin-like growth factor-1 (IGF1) protects the survival of neurons and glia cells, stimulates the regeneration of myelin and promotes proliferation and differentiation of glia cells (9). It can also attenuate the damage of the blood-brain barrier (BBB) and alleviate immune-mediated inflammation (10, 11). Low levels of serum IGF1 in serum were demonstrated to be associated with susceptibility to MS (12), and were also associated with cognitive impairment and fatigue in MS (13). The bioavailability of IGF1 is regulated by insulin-like growth factor binding proteins (IGFBP). IGFBP3 is the most abundant IGFBP in human serum (14). Rather than controlling IGF activity, IGFBP3 can directly inhibit cell growth (15). In several studies, decreased levels of IGFBP3 and reduced bioavailability of IGF1 were reported in the serum of MS patients (16, 17). Vascular endothelial growth factor (VEGF), also called vascular permeability factor, mediates endothelial-specific mitogenesis, increases capillary permeability, and contributes to BBB breakdown (18). Additionally, VEGF induces major histocompatibility complex (MCH I & II) expression in the brain, and is a chemo attractant to monocytes (19). Upregulation of VEGF was detected in serum and central nervous tissue in MS patients (20).

We hypothesize that growth factors have essential function in the initiation of MS, thus establishing the causal relationship between circulating levels of growth factors and MS risk is important from clinical perspective. However, confined by methodological defects (such as residual confounding and reverse causality), traditional observational studies are unable to ascertain the causal relationships between exposures and corresponding diseases (21). Mendelian randomization (MR) is a method to exploit causality by using genetic variants as proxies (instrumental variables) to predict the effect of the exposure on disease risk (22). Since the assortment of alleles at meiosis is random and germline genetic variants are fixed at conception, MR studies are unaffected by the disease process and can avoid confounding and reverse causality.

Leveraging up-to-date genome-wide association studies (GWASs), we conducted a two-sample MR analysis to detangle the potential causal roles of growth factors on MS risk in this study.

## Materials and Methods

**Figure 1:**
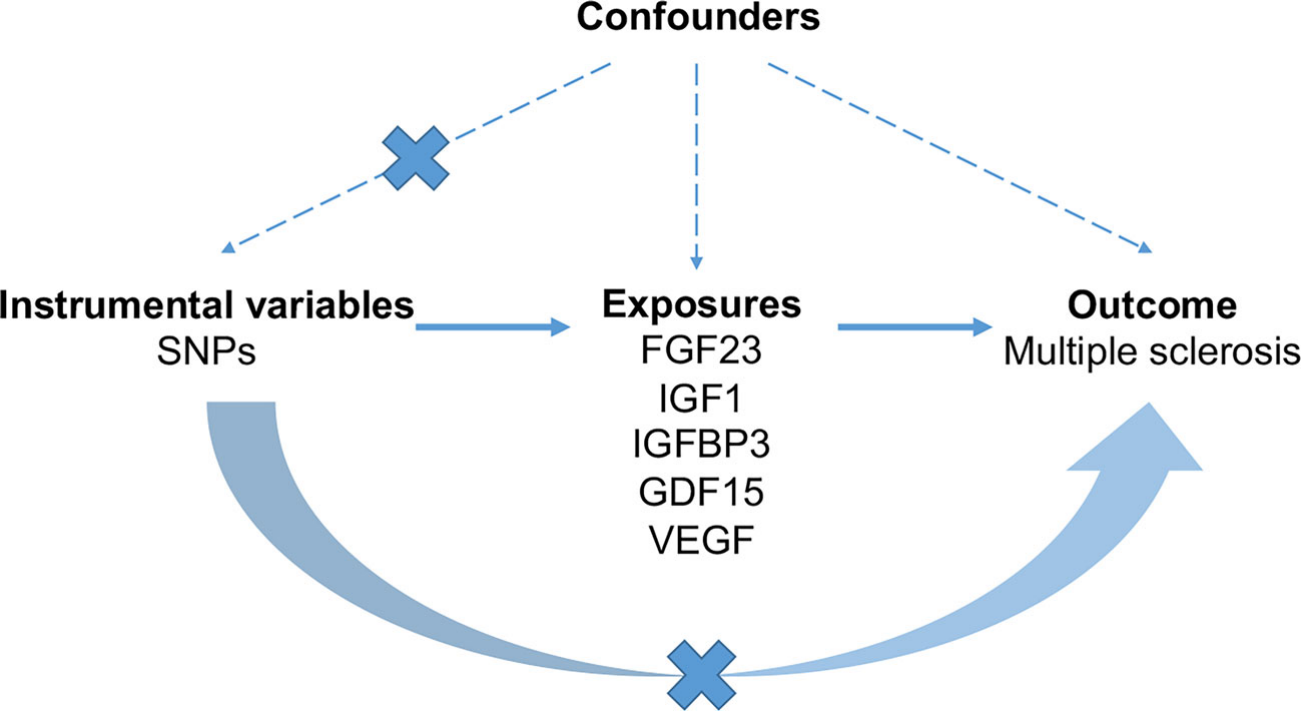
The schematic diagram demonstrating concept of the MR design. Three key assumptions underlay standard selection procedure of instrumental SNPs. First, selected SNPs were robustly associated (*p* < 5 × 10^-8^) with exposures of interest. Second, later-life confounders of the exposure-outcome link scarcely existed, given that genetic variants were inherited at gamete formation when randomized allocation of instrumental variant alleles among the large population were determined. Thirdly, the exclusion-restriction assumption, that instrumental SNPs affected the outcome only though the exposure, were examined by sensitivity analyses. FGF23, fibroblast growth factor 23; GDF15, growth differentiation factor 15; IGF1, insulin-like growth factor 1; IGFBP3, insulin-like growth factor-binding protein 3; VEGF, vascular endothelial growth factor; MS, multiple sclerosis; SNP, Single-nucleotide polymorphism.

Based on public summary-level data derived from GWAS, we conducted a two-sample MR study to investigate the causal association of serum levels of FGF23, GDF1, IGF1, IGFBP3 and VEGF with MS (**Figure 1**). No additional consent from participants or ethical approval was required other than what had been completed in prior studies.

Genetic instruments for FGF23 were extracted from a meta-analysis of GWAS conducted by the ReproGen Alliance, consisting of 7 studies with 16,624 European participants (the mean age was ranged from 36.4 to 78.0 years old and 54.5% were women) (23). The GWAS data of FGF23 were adjusted for sex, age and top ten components of ancestry in linear regression (23). A meta-analysis of GWAS consisting 4 community-based cohorts with 5440 individuals of European ancestry (the mean age was 62 years and 53% were women) was utilized to obtain GDF15 genetic associations (24). Genetic instruments for serum IGF1 levels were selected from a GWAS of 451,993 European-descent individuals (the mean age was 56.5 years and 54% were women) in UK Biobank repository (25). Effect estimates for SNPs associated with IGFBP3 were obtained from a meta-analysis including 13 studies with up to 18, 995 individuals (8053 men and 10 942 women) (26). Genetic predictors of VEGF were obtained from a meta-analysis of GWAS including 16, 112 individuals (the mean age was 54.8 years and 54% were women) (27).

We obtained 7, 5, 318, 4 and 10 instrumental variables for FGF23 (23), GDF15 (24), IGF1 (25), IGFBP3 (26) and VEGF (27), respectively. All instrumental SNPs were strongly associated with the above circulating growth factors (*p* < 5 × 10^-8^). We checked linkage disequilibrium (*r^2^* > 0.01 within 1Mb window) between instrumental SNPs with 1000 Genomes EUR reference panel. Summary statistics of MS were retrieved from a recent GWAS (28) conducted by the International Multiple Sclerosis Genetics Consortium in 14,802 cases and 26,703 healthy controls of the European descent (**Supplementary Table 1**). For Instrumental variables which were not present in summary statistics of MS, proxied SNPs (*r^2^* ≥ 0.8) were utilized if available. Effect estimates of palindromic variants were directly utilized since datasets downloaded from the GWAS Catalog were harmonized in terms of the forward strand. Since odds ratio (OR) was commonly reported for dichotomized traits, we made further harmonization, that is, OR in summary statistics of MS underwent log-transformation to get log-OR, which was equivalent to beta for continuous exposures. Demographic information of participants was provided in detail in original studies. The exposure and outcome datasets were merged with regard to each instrumental SNP and its effect allele, and the harmonized datasets (**Supplementary Tables 2–6**) were subject for ensuing analyses.

We conducted MR analyses using the TwoSampleMR package (29) in the R 3.6.1 software. Causal estimate by each instrumental variable SNP_k_ can be derived by dividing its effect on the outcome *Y_k_* by its effect on the exposure *X_k_*, that is, the Wald ratio *Y_k_*/*X_k_* and the associated standard error σ*Y_k_*/*X_k_*. To combine causal estimates from multiple SNPs, the inverse-variance-weighted (IVW) method was employed as the primary approach (30), with the causal estimate ${\hat{\beta }}_{IVW}$ and related standard error ${\hat{\sigma }}_{IVW}$ given by two formulae:


$${\hat{\beta }}_{IVW}=\frac{\Sigma \;X_{k}Y_{k}\sigma_{Y_{k}}^{-2}}{\Sigma \;X_{k}^{2}\sigma_{Y_{k}}^{-2}}$$

$${\hat{\sigma }}_{IVW}=\sqrt{\frac{1}{\Sigma \;X_{k}^{2}\sigma_{Y_{k}}^{-2}}}$$

Two complementary approaches, weighted median and MR-Egger were also conducted (31, 32), since IVW estimates would be biased if not all instrumental variables were valid. Weighted median estimator was based on the relaxed assumption that more than 50% of variants were valid (32). MR-Egger regression was capable of identifying and adjusting for unbalanced horizontal pleiotropy by the regression intercept and slope, respectively (31). Forest plots were presented to visualize MR results, where causal estimates on the risk of MS were reported in OR and related confidence intervals (CI) in terms of one standard deviation increase in circulating levels of growth factors. Scatter plots and leave-one-out plots were depicted to examine the robustness of primary MR results. Bonferroni-corrected significance threshold at *p* < 0.05/5 was utilized.

## Results

### MR Analysis of FGF23 on the Risk of MS

**Figure 2:**
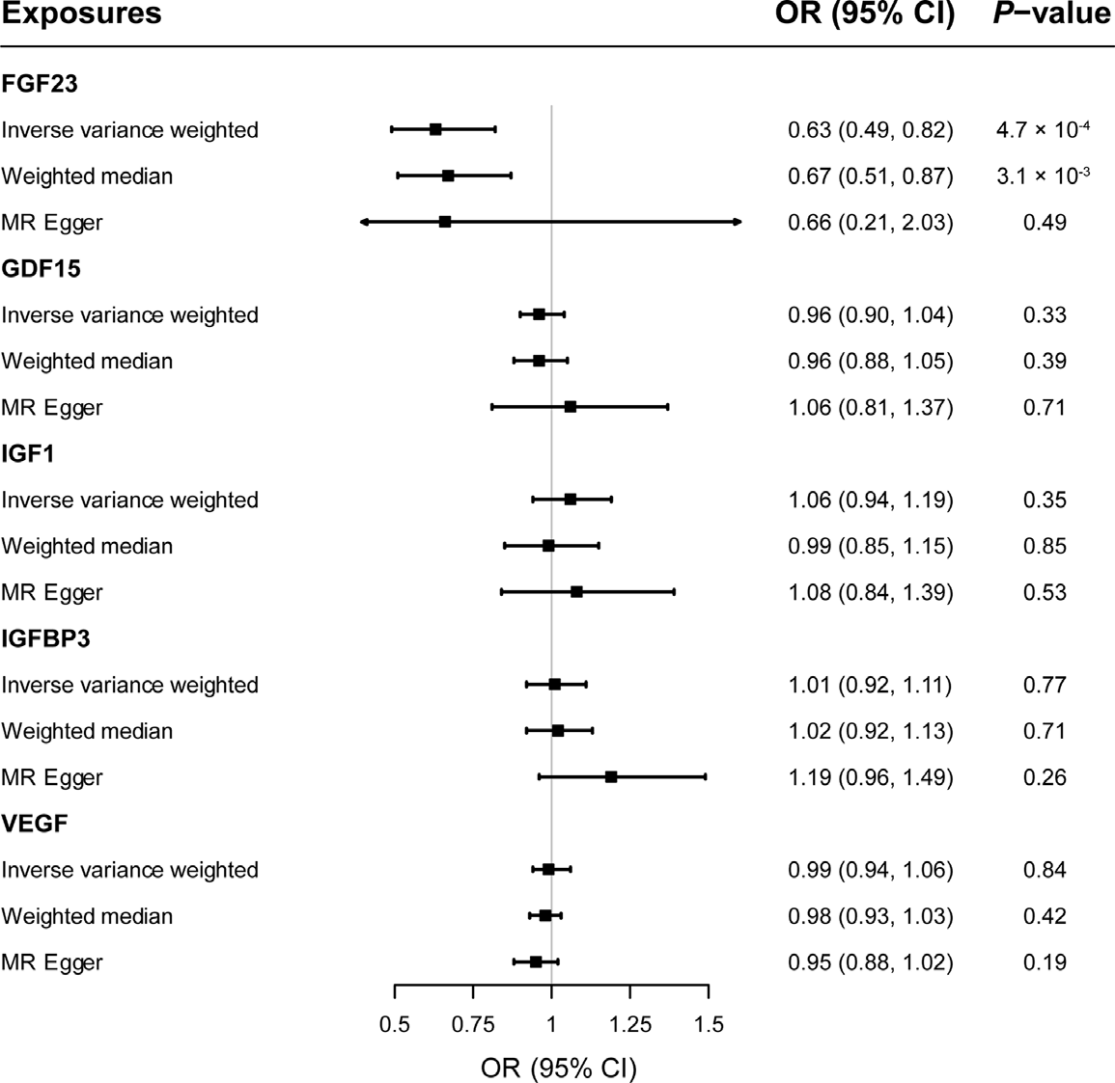
The forest plot delineating causal estimates of growth factors on multiple sclerosis. CI, confidence interval; FGF23, fibroblast growth factor 23; GDF15, growth differentiation factor 15; IGF1, insulin-like growth factor 1; IGFBP3, insulin-like growth factor-binding protein 3; VEGF, vascular endothelial growth factor; MS, multiple sclerosis; SNP, Single-nucleotide polymorphism; OR, odds ratio.

**Figure 3:**
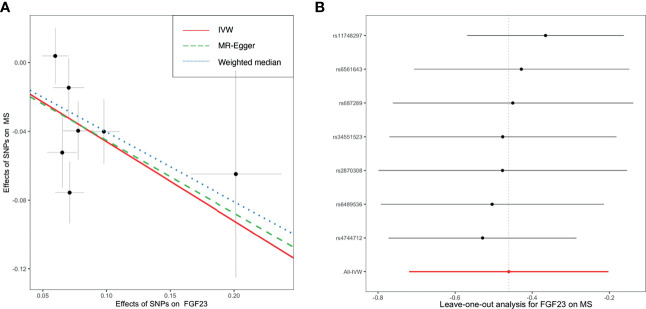
The scatter plot **(A)** and leave-one-out plot **(B)** in the Mendelian randomization analysis of circulating FGF23 on the risk of MS. FGF23, fibroblast growth factor 23; IVW, inverse variance weighted; MS, multiple sclerosis; SNP, Single-nucleotide polymorphism.

As shown in **Figure 2**, primary MR analysis by the IVW method showed that circulating levels of FGF23 affected the risk of MS (OR = 0.63; 95% CI, 0.49-0.82; *p* = 4.7 × 10^-4^). Weighted median estimators also suggested that FGF23 was associated with lower MS risk (OR = 0.67; 95% CI, 0.51-0.87; *p* = 3.1 × 10^-3^). Notably, by the MR-Egger approach assessing the causal effect of FGF23 on MS, there was no evidence of horizontal pleiotropy (intercept = -0.003, *p* = 0.95), but the causal estimate (OR = 0.66) was accompanied by a wide 95% CI (0.21-2.03), indicating a comprised power (*p* = 0.49). In the scenario, we considered primarily the causal estimate by the IVW approach, and the effect of FGF23 on MS was deemed significant. After examining the scatter plot and leave-one-out plot (**Figure 3**), there was no evidence supporting the existence of outlier SNPs, indicating negligible heterogeneity among all instrumental variants.

### Causal Estimates of GDF15, IGF1, IGFBP3, and VEGF on MS

Overall, genetically predicted concentrations of GDF15, IGF1, IGFBP3 and VEGF were not associated with the risk of MS. Primary MR results (**Figure 2**) demonstrated that there was no causal relationship between GDF15 and MS (OR = 0.96; 95% CI, 0.90-1.04; *p* = 0.33); neither did the causal effect of IGF1 (OR = 1.06; 95% CI, 0.94-1.19; *p* = 0.35), IGFBP3 (OR = 1.01; 95% CI, 0.92-1.11; *p* = 0.77), or VEGF (OR = 0.99; 95% CI, 0.94-1.06; *p* = 0.84) on MS reach nominal significance. Likewise, two additional MR methods, weighted median and MR-Egger gave similar causal estimates (all *p* > 0.05). Scatter plots (**Supplementary Figure 1**) and leave-one-out plots (**Supplementary Figure 2**) indicated no presence of outlying instrumental variables, which would exert unproportionate effects on the MR estimates otherwise.

## Discussion

In this MR study, we found that genetically predicted decreased circulating FGF23 levels may be associated with increased risk of MS. Meanwhile, we didn’t find any causal relationship between circulating levels of GDF15, IGF1, IGFBP3 and VEGF and MS risk.

Primarily, FGF23 leads to decreased levels of phosphate, 1,25-dihydroxyvitamin D and parathyroid hormone in circulation (33, 34). Low vitamin D levels and low sun exposure have been generally recognized as risk factors for MS (35). The klotho–FGF23–vitamin D axis is fundamental in the regulation of calcium and phosphorus metabolisms. In addition, FGF23 is also secreted from neurons or the choroid plexus which can disrupt the integrity of BBB and alter the phosphate metabolism in cerebrospinal fluid (33). So it was postulated that the disequilibrium of FGF23 was associated with MS. But observational studies showed inconsistent results. A recent cohort study including 91 MS patients didn’t find any difference (*p* = 0.65) between MS patient and healthy controls in plasma FGF23 concentrations (36). Another cohort study including 14 relapsing-remitting MS (RRMS) patients also found that FGF23 concentrations in MS patients were comparable to controls (*p* = 0.59) (37). While a previous observational study including 32 RRMS patients found overexpression of FGF23 in MS patients (*p* < 0.01) and an association of high FGF23 levels (approximately 2.5-fold higher) with comorbidities such as cardiovascular diseases in MS (33). Stein et al. revealed a disequilibrium of the PTH-FGF23-vitamin D axis in RRMS, with higher plasma FGF23 in winter (*p* = 0.04) and comparable FGF23 levels in summer (*p* = 0.14) (38). The discrepancies of these observational studies might be caused by confounding, reverse causation, and selection bias et al.

Interestingly, although FGF23 was regarded as a negative regulator of calcitriol biosynthesis, we found that genetically predicted FGF23 was inversely associated with risk of MS in this study. Similar to our result, Aleagha et al. also reported a significant negative correlation between FGF23 in cerebrospinal fluid and the Expanded Disability Status Scale of patients with RRMS (37). The result of a recent MR study suggested no strong evidence for the association between FGF23 and serum levels of 25-hydroxyvitamin D (p = 0.28) or calcium (p = 0.37) (39). So we hypothesize that the potential protective effect of FGF23 on MS is probably not *via* vitamin D pathways. The contradictory results by Ellidag et al. (33) and Stein et al. (38) might be caused by the effects of treatments in RRMS patients since previous studies have indicated that the expression of FGF23 might be influenced by medication (40).

Since only a few SNPs were available for FGF23, verification of our result with larger GWAS data with more genetic instruments (including rare variants) is needed. And more basic investigations are required to elucidate the underlying mechanisms related to the effect of FGF23 on MS. Our result also implied a potential therapeutic role of FGF23 for the treatment of MS. Since our study adopted serum FGF23, FGF23 might not need to cross BBB to exert a therapeutic effect and peripheral use of FGF23 might be helpful (41). Further clinical trials are warranted to explore the potential therapeutic effects of FGF23 in MS patients.

The main strength of our study is leveraging large-sample genetic data from several sources to clarify causal relationships between growth factors and MS. For these traits, we utilized up-to-date and largest GWASs as of December 31, 2020 in this study. Summary statistics of IGF1 were released recently, the GWAS incorporated ~467,000 participants from UK Biobank which has been the largest cohort worldwide. However, samples sizes of the other four exposures were relatively restricted, especially for GDF15; accordingly, number of instrumental variables at genome-wide significance and variance explained for concerned exposures by them were largely limited. In this scenario, we would have insufficient power to detect weak causal effects, and thus should be cautious with the strength of evidence provided by this study. It has been known that with the sample size increasing, more significant loci will be identified by GWASs. Last two decades have witnessed great advancement in sequencing technology and their availability and affordability. GWASs in larger populations can be expected in the future, and once summary statistics available, it is necessary to update these MR analyses to get new findings. Phenotypic observational studies cannot avoid the bias from confounding variables and reverse causation, thus spurious interplay between exposure and diseases may arise (42). Randomized controlled trials (RCTs) are less influenced by such defects of observational studies, but large-scale RCTs are very expensive. MR is a powerful method to infer the causality and could enable more robust understandings of causal molecular biology (41). Besides, MR imitates RCTs since genetic variants were randomly allocated at conception (43). And SNPs mimic lifelong exposure to medications which makes MR the nature’s RCTs (43). Meanwhile, MR studies are effectively blind (41). It is proposed that drugs with genetic evidence might be twice likely to proceed from Phase I to approval (44). So MR is an economic and efficient way to screen possible drug targets. Leveraging population level data, we provide genetic support for prioritizing FGF23 as a potential treatment for MS.

Although MR provides reliable evidence of causation, it cannot replace RCTs as the best approach evaluating drug efficacy. Rather than disease progression, GWASs pertain to disease risk. The effects of MR instruments mimic low-dose exposure across the entire life-course and usually have smaller effects, whereas drugs are prescribed later in life and generally have larger effects (45). So MR is more suitable to study public health policies or preventative interventions, and less in predicting the outcome of RCTs which only last years and measure progression (41). Secondly, given largely unknown aspects of human genomes, MR studies are subject to the presence of linkage disequilibrium, cryptic relatedness, genetic heterogeneity, pleiotropy, canalization or co-variable adjustment. Thirdly, MR is unable to assess potential non-linear relationships between risk factors and MS. Fourthly, our GWAS data were sampled from European ancestry populations. While MR minimizes the risk of population stratification and false-positive GWAS signals, heterogeneity across different populations limits generalization of the results to other populations with different genetic backgrounds. Lastly, the effect of FGF23 on MS might be sex specific, but sex-specific analysis cannot be implemented due to lack of relevant genetic summary statistical data.

In conclusion, our MR results supported a potential causality between decreased FGF23 levels and higher risk of MS.

## Data Availability Statement

The original contributions presented in the study are included in the article/**Supplementary Material**. Further inquiries can be directed to the corresponding author.

## Author Contributions

HL: study design, funding acquisition, literature research, data acquisition and manuscript preparation. P-FW: data analysis and statistical analysis. D-LM: manuscript editing and manuscript revision. WZ: manuscript editing and manuscript revision. MS: manuscript editing. All authors contributed to the article and approved the submitted version.

## Funding

This work was supported by scientific research and cultivation plan of Beijing Municipal Hospital (grant PX2021036).

## Conflict of Interest

The authors declare that the research was conducted in the absence of any commercial or financial relationships that could be construed as a potential conflict of interest.

## Publisher’s Note

All claims expressed in this article are solely those of the authors and do not necessarily represent those of their affiliated organizations, or those of the publisher, the editors and the reviewers. Any product that may be evaluated in this article, or claim that may be made by its manufacturer, is not guaranteed or endorsed by the publisher.
