# Supplementary material for: Growth Factors and Their Roles in Multiple Sclerosis Risk
Source: Front Immunol. 2021 Oct 21;12:768682. doi: 10.3389/fimmu.2021.768682 (PMC8566812; doi:10.3389/fimmu.2021.768682)
Supplement: Supplementary file 1 [file DataSheet_1.pdf]

Supplementary Material

Supplementary Table 1. Basic information for summary-level datasets utilized in the Mendelian randomization study

Supplementary Table 2. Summary statistics utilized in the Mendelian randomization study of FGF-23 on MS

Supplementary Table 3. Summary statistics utilized in the Mendelian randomization study of GDF-15 on MS

Supplementary Table 4. Summary statistics utilized in the Mendelian randomization study of IGF-1 on MS

Supplementary Table 5. Summary statistics utilized in the Mendelian randomization study of IGFBP-3 on MS

Supplementary Table 6. Summary statistics utilized in the Mendelian randomization study of VEGF on MS

Supplementary Figure 1. Scatter plots in the Mendelian randomization analyses.

Supplementary Figure 2. Leave-one-out plots in the Mendelian randomization analyses.

Supplementary Table 1. Basic information for summary-level datasets utilized in the Mendelian randomization study

Phenotype	Author and Year	Sample Size	SNPs	Population	PubMed ID
FGF23	Robinson-Cohen et al., 2018		7	European	30217807
GDF15	Jiang et al., 2018	5,440	5	European	29628937
IGF1	Zanetti et al., 2020	467,066	318	European	33125266
IGFBP3	Teumer et al., 2016	30, 884	4	European	27329260
VEGF	Choi SH et al., 2016	16, 112	10	European	26910538
Multiple Sclerosis	Patsopoulos et al., 2018	14,802 / 26,703	–	European	31604244

Abbreviations: FGF23, fibroblast growth factor 23; GDF15, growth differentiation factor 15; IGF1, insulin-like growth factor 1; IGFBP3, insulin-like growth factor-binding protein 3; VEGF, vascular endothelial growth factor; MS, multiple sclerosis.

Supplementary Table 2. Summary statistics utilized in the Mendelian randomization study of FGF-23 on MS

SNP	Coordinate (GRCh37)	Gene	EA/OA	Association with FGF-23			Association with MS		
				Beta	Se	*P*-value	Beta	Se	*P*-value
rs2870308	20:52727953	CYP24A1	A/C	-0.098	0.013	4.70×10^{-15}	0.04	0.019	0.033
rs687289	9:136137106	ABO	A/G	0.078	0.012	5.45×10^{-11}	-0.04	0.017	0.020
rs11748297	5:176800361	SLC34A1	A/G	-0.071	0.011	3.57×10^{-10}	0.076	0.018	0.00002
rs6489536	12:4491909	FGF23	C/G	0.07	0.012	7.77×10^{-9}	-0.015	0.017	0.399
rs4744712	9:71434707	PIP5K1B	A/C	-0.06	0.01	1.08×10^{-8}	-0.004	0.016	0.817
rs34551523	5:146629145	STK32A	A/G	0.202	0.036	1.82×10^{-8}	-0.065	0.06	0.281
rs6561643	13:33509079	KL	A/T	-0.065	0.012	3.18×10^{-8}	0.052	0.02	0.011

Note: FGF-23, fibroblast growth factor 23; MS, multiple sclerosis; SNP, Single-nucleotide polymorphism; EA, effect allele; OA, other allele; Beta (Se) represents coefficients by the additive regression model, and Beta > 0 denotes an additional copy of effect allele can increase serum FGF-23 levels or MS risk.

Supplementary Table 3. Summary statistics utilized in the Mendelian randomization study of GDF-15 on MS

SNP	Coordinate (GRCh37)	Gene	EA/OA	Association with GDF-15			Association with MS		
				Beta	Se	*P*-value	Beta	Se	*P*-value
rs888663	19:18484922	PGPEP1	T/G	0.303	0.024	2.64×10^{-35}	-0.018	0.022	0.415
rs1227731	19:18497903	GDF15	A/G	0.309	0.026	3.37×10^{-33}	-0.006	0.025	0.797
rs749451	19:18479647	PGPEP1	C/T	0.218	0.019	2.54×10^{-31}	0.008	0.017	0.652
rs3195944	19:18476711	PGPEP1	G/A	0.334	0.029	2.39×10^{-30}	-0.015	0.027	0.590
rs17725099	19:18482358	PGPEP1	A/G	0.135	0.025	4.13×10^{-8}	-0.032	0.02	0.115

Note: GDF-15, growth differentiation factor-15; MS, multiple sclerosis; SNP, Single-nucleotide polymorphism; EA, effect allele; OA, other allele.

Supplementary Table 4. Summary statistics utilized in the Mendelian randomization study of IGF-1 on MS

SNP	Coordinate (GRCh37)	Gene	EA/OA	Association with IGF-1			Association with MS		
				Beta	Se	*P*-value	Beta	Se	*P*-value
rs856540	7:46708387	TNS3	A/G	-0.106	0.002	1.00×10^{-200}	0.002	0.018	0.929
rs3842763	11:2179204	INS-IGF2	T/G	-0.084	0.003	1.37×10^{-192}	0.001	0.025	0.981
rs11111274	12:102838128	IGF1	G/A	0.075	0.003	1.75×10^{-172}	0.019	0.018	0.300
rs2184968	6:126760994	CENPW	C/T	0.065	0.002	6.12×10^{-164}	-0.028	0.016	0.082
rs1260326	2:27730940	GCKR	T/C	-0.060	0.002	4.94×10^{-134}	-0.009	0.017	0.577
rs165316	1:91533297	ZNF644	G/A	0.070	0.003	2.78×10^{-119}	0.037	0.022	0.091
rs125124	7:130584684	KLF14	G/C	0.057	0.003	8.99×10^{-107}	-0.012	0.018	0.501
rs572169	3:172165727	GHSR	T/C	0.054	0.003	1.66×10^{-99}	-0.016	0.018	0.373
rs12749024	1:176522365	PAPPA2	T/C	0.071	0.003	4.41×10^{-98}	-0.021	0.024	0.392
rs9738365	12:31997635	H3F3C	A/C	0.056	0.003	5.94×10^{-95}	-0.029	0.018	0.112
rs1800574	12:121416864	HNF1A	T/C	0.140	0.007	8.44×10^{-87}	0.184	0.059	0.002
rs139974673	15:44027885	PDIA3	C/T	-0.146	0.008	1.82×10^{-80}	-0.049	0.118	0.681
rs2854746	7:45960645	IGFBP3	C/G	-0.046	0.002	4.53×10^{-78}	0.025	0.017	0.151
rs11644716	16:1862927	HAGH	C/T	0.085	0.005	5.54×10^{-76}	-0.018	0.032	0.580
rs2153960	6:108988184	FOXO3	G/A	-0.048	0.003	8.23×10^{-75}	0.016	0.018	0.372
rs35668185	5:168256455	SLIT3	C/T	-0.054	0.003	6.23×10^{-74}	0.017	0.02	0.404
rs7261425	20:20068635	CFAP61	G/C	-0.048	0.003	1.47×10^{-71}	0.004	0.018	0.828
rs1832007	10:5254847	AKR1C4	G/A	0.053	0.003	5.60×10^{-59}	-0.010	0.023	0.663
rs6082354	20:21217976	KIZ	A/C	0.041	0.003	1.13×10^{-58}	0.018	0.017	0.296
rs4709995	6:166313447	PDE10A	T/C	0.039	0.002	4.21×10^{-57}	0.007	0.016	0.650
rs8105174	19:10347032	S1PR2	T/C	-0.048	0.003	1.03×10^{-54}	0.013	0.022	0.564
rs17597773	1:221054761	HLX	G/C	-0.043	0.003	4.25×10^{-54}	-0.001	0.019	0.937
rs7178424	15:62380259	C2CD4A	T/C	-0.037	0.002	8.43×10^{-54}	0.016	0.017	0.328

rs62229260	21:37437566	SETD4	G/A	-0.037	0.002	1.47×10^{-51}	-0.034	0.017	0.047
rs55681913	5:42687629	GHR	C/T	0.058	0.004	4.94×10^{-51}	-0.001	0.048	0.988
rs7131605	11:48248097	PTPRJ	A/T	0.040	0.003	1.08×10^{-47}	0.033	0.019	0.078
rs4234798	4:7219933	SORCS2	T/G	-0.036	0.002	1.16×10^{-47}	0.023	0.017	0.170
rs263062	19:5022058	KDM4B	C/T	-0.037	0.003	1.74×10^{-46}	0.021	0.018	0.251
rs151196451	5:59028725	PDE4D	[illegible]ACTTTCAC[illegible]	-0.037	0.003	5.33×10^{-46}	-0.020	0.017	0.243
rs520829	6:160767905	SLC22A3	G/T	-0.033	0.002	5.07×10^{-44}	-0.004	0.017	0.819
rs7662792	4:45121873	GNPDA2	A/T	0.035	0.003	2.76×10^{-43}	-0.002	0.017	0.904
rs34495733	7:6763641	ZNF12	A/G	-0.047	0.003	1.35×10^{-42}	0.006	0.024	0.803
rs6822348	4:100053894	ADH4	A/T	-0.036	0.003	1.43×10^{-42}	-0.006	0.018	0.746
rs687339	3:135932359	MSL2	C/T	-0.039	0.003	8.11×10^{-42}	-0.009	0.02	0.648
rs7632381	3:141106063	ZBTB38	C/T	-0.032	0.002	4.93×10^{-41}	-0.055	0.016	0.001
rs10171272	2:25946636	ASXL2	A/C	0.035	0.003	2.86×10^{-40}	0.023	0.018	0.212
rs114165349	1:27021913	ARID1A	C/G	-0.105	0.008	3.59×10^{-40}	0.051	0.059	0.391
rs12659034	5:137773525	KDM3B	T/C	0.039	0.003	2.18×10^{-39}	0.017	0.02	0.391
rs3752416	6:26045929	HIST1H3C	T/C	0.034	0.003	1.66×10^{-38}	-0.045	0.017	0.009
rs9532512	13:40769897	FOXO1	A/G	0.040	0.003	3.80×10^{-38}	0.029	0.021	0.165
rs59502288	16:31109287	VKORC1	[illegible]TCATCCA/[illegible]	-0.032	0.003	6.11×10^{-38}	-0.044	0.017	0.009
rs73271090	5:132313550	AFF4	A/G	-0.040	0.003	1.28×10^{-36}	-0.017	0.022	0.438
rs456179	9:4851966	RCL1	C/T	-0.044	0.004	3.00×10^{-33}	0.027	0.025	0.279
rs6940544	6:88000581	GJB7	A/G	-0.029	0.002	3.40×10^{-33}	-0.071	0.016	0.000
rs147400256	16:1942182	HS3ST6	A/C	-0.102	0.009	1.12×10^{-32}	0.009	0.079	0.910
rs12979891	19:49227256	RASIP1	C/T	0.028	0.002	1.37×10^{-31}	0.058	0.017	0.001
rs6519133	22:39096602	JOSD1	C/T	-0.028	0.002	6.16×10^{-31}	0.004	0.018	0.834
rs8059803	16:81603001	CMIP	G/A	-0.029	0.003	1.80×10^{-29}	-0.021	0.019	0.253
rs4782568	16:83980529	OSGIN1	G/C	-0.027	0.002	6.58×10^{-29}	-0.022	0.021	0.284
rs7314285	12:111522026	CUX2	G/T	0.051	0.005	2.15×10^{-27}	0.041	0.034	0.229
rs951740	1:44011737	PTPRF	G/A	0.027	0.002	3.88×10^{-27}	-0.009	0.017	0.609

rs8126001	20:62711459	OPRL1	T/C	0.026	0.002	9.93×10^{-27}	-0.007	0.018	0.709
rs583104	1:109821307	PSRC1	G/T	0.030	0.003	1.10×10^{-26}	-0.024	0.02	0.228
rs7676961	4:39684217	UBE2K	T/C	0.025	0.002	1.37×10^{-26}	0.023	0.016	0.163
rs62280667	3:101084604	SENP7	C/T	0.027	0.003	2.64×10^{-26}	0.008	0.018	0.648
rs7953987	12:98167068	TMPO	A/G	0.026	0.002	8.15×10^{-26}	0.003	0.017	0.876
rs1886274	10:102651076	SLF2	C/G	-0.025	0.002	8.58×10^{-26}	0.007	0.016	0.660
rs9272632	6:32608180	HLA-DQA1	G/A	-0.029	0.003	5.85×10^{-25}	-0.162	0.063	0.010
rs7517340	1:243710190	AKT3	T/C	-0.032	0.003	8.92×10^{-25}	0.005	0.022	0.802
rs2856321	12:11855773	ETV6	G/A	0.025	0.002	3.28×10^{-24}	-0.033	0.017	0.051
rs3826331	17:38150492	PSMD3	T/C	-0.025	0.002	3.89×10^{-24}	-0.006	0.017	0.716
rs9611567	22:41769754	TEF	G/A	-0.028	0.003	7.89×10^{-24}	0.026	0.019	0.176
rs1349852	4:69533217	UGT2B15	C/A	-0.024	0.002	8.46×10^{-24}	-0.019	0.021	0.372
rs3936674	6:152167311	ESR1	A/G	0.025	0.003	8.83×10^{-24}	0.018	0.018	0.319
rs5742915	15:74336633	PML	C/T	0.024	0.002	1.22×10^{-23}	0.018	0.017	0.286
rs28803639	3:138888291	PRR23C	T/G	-0.026	0.003	1.59×10^{-23}	0.004	0.018	0.825
rs113441031	16:69763280	NQO1	T/C	-0.031	0.003	1.06×10^{-22}	0.019	0.022	0.397
rs1150974	12:32050598	KIAA1551	A/C	0.037	0.004	1.30×10^{-22}	-0.006	0.027	0.829
rs9297994	8:59392324	CYP7A1	G/A	0.024	0.003	2.11×10^{-22}	0.011	0.017	0.534
rs17429745	4:106038169	TET2	T/G	-0.025	0.003	2.21×10^{-22}	-0.043	0.017	0.014
rs1127313	1:154556425	ADAR	A/G	-0.023	0.002	7.15×10^{-22}	0.009	0.016	0.579
rs9829214	3:101258135	TRMT10C	T/C	0.024	0.002	8.25×10^{-22}	0.011	0.018	0.528
rs35958394	19:10881250	DNM2	C/G	-0.024	0.003	8.28×10^{-22}	-0.037	0.017	0.032
rs3127580	6:160710851	SLC22A2	T/C	0.031	0.003	1.11×10^{-21}	-0.013	0.024	0.583
rs2737205	8:116610180	TRPS1	T/C	0.023	0.002	1.94×10^{-21}	0.023	0.016	0.159
rs112293610	7:14226261	DGKB	C/A	-0.025	0.003	2.67×10^{-21}	-0.007	0.018	0.696
rs72745405	9:96246180	FAM120A	T/C	0.029	0.003	1.11×10^{-20}	0.037	0.025	0.139
rs17050272	2:121306440	INHBB	A/G	-0.022	0.002	1.45×10^{-20}	0.004	0.017	0.816
rs2853946	6:31247203	HLA-C	T/A	0.024	0.003	1.61×10^{-20}	-0.119	0.018	0.000

rs2274224	10:96039597	PLCE1	C/G	-0.022	0.002	2.76×10^{-20}	0.012	0.017	0.469
rs78509281	9:109566543	ZNF462	T/C	0.050	0.006	3.19×10^{-20}	0.025	0.057	0.658
rs71211295	15:99177655	IGF1R	C/CG	-0.023	0.002	3.64×10^{-20}	-0.023	0.017	0.175
rs876375	14:101204319	DLK1	A/G	0.022	0.002	3.87×10^{-20}	-0.015	0.017	0.363
rs74327681	18:74981730	GALR1	T/C	-0.054	0.006	5.59×10^{-20}	-0.070	0.039	0.076
rs41303235	9:4985388	JAK2	T/C	0.053	0.006	6.00×10^{-20}	-0.023	0.033	0.491
rs4270941	8:134589010	ST3GAL1	T/C	-0.022	0.002	6.64×10^{-20}	-0.007	0.017	0.703
rs1351394	12:66351826	HMGA2	T/C	-0.022	0.002	7.37×10^{-20}	0.002	0.017	0.885
rs1994147	15:95829524	MCTP2	A/T	0.024	0.003	1.24×10^{-19}	0.039	0.021	0.065
rs35814008	7:73055461	MLXIPL	C/A	0.032	0.004	1.39×10^{-19}	0.020	0.03	0.510
rs13301073	9:128284378	MAPKAP1	A/G	-0.022	0.002	1.69×10^{-19}	-0.031	0.017	0.069
rs17487338	3:138848435	PRR23C	A/G	-0.029	0.003	2.84×10^{-19}	0.032	0.039	0.419
rs33912345	14:60976537	SIX6	C/A	0.022	0.002	4.43×10^{-19}	-0.004	0.017	0.825
rs484084	1:234857676	IRF2BP2	T/C	-0.021	0.002	8.95×10^{-19}	0.023	0.016	0.162
rs998075	6:160468278	IGF2R	G/A	-0.021	0.002	1.34×10^{-18}	0.004	0.017	0.815
rs12952818	17:17988591	DRG2	G/A	0.022	0.002	1.48×10^{-18}	0.001	0.017	0.945
rs34935748	3:138865561	PRR23C	G/T	0.022	0.003	2.19×10^{-18}	0.002	0.017	0.899
rs13178887	5:88355993	MEF2C	C/T	-0.021	0.002	4.33×10^{-18}	-0.049	0.017	0.004
rs74774288	16:5922263	RBFOX1	T/G	-0.026	0.003	6.02×10^{-18}	-0.012	0.021	0.583
rs11642090	16:81730582	CMIP	C/T	-0.021	0.002	6.45×10^{-18}	0.021	0.018	0.221
rs57267144	2:203392479	BMPR2	C/G	-0.023	0.003	8.06×10^{-18}	0.008	0.019	0.693
rs17400325	2:178565913	PDE11A	C/T	0.051	0.006	1.00×10^{-17}	0.032	0.042	0.454
rs10657263	11:49690460	OR4C13	C/G	0.020	0.002	1.01×10^{-17}	0.048	0.017	0.006
rs11024614	11:18326758	HPS5	C/T	0.021	0.002	1.38×10^{-17}	0.009	0.018	0.622
rs667668	5:134597890	C5orf66	A/G	-0.020	0.002	1.41×10^{-17}	0.023	0.017	0.188
rs12702434	7:1195796	ZFAND2A	C/T	0.021	0.002	1.98×10^{-17}	0.001	0.018	0.970
rs13379043	14:74250126	ELMSAN1	C/T	-0.023	0.003	3.11×10^{-17}	-0.006	0.019	0.729
rs3775288	4:46391821	GABRA2	G/A	-0.030	0.004	3.42×10^{-17}	-0.028	0.033	0.404

rs174554	11:61579463	FADS1	G/A	-0.021	0.003	4.77×10^{-17}	-0.011	0.017	0.520
rs6088579	20:33284624	TP53INP2	A/G	-0.026	0.003	8.12×10^{-17}	-0.019	0.022	0.395
rs10405357	19:54759666	LILRB5	C/T	-0.020	0.002	1.02×10^{-16}	-0.006	0.021	0.763
rs2992073	1:26249835	STMN1	C/G	0.023	0.003	1.32×10^{-16}	0.002	0.019	0.918
rs62182127	2:219279588	VIL1	G/A	-0.020	0.002	1.47×10^{-16}	0.021	0.016	0.208
rs7628689	3:88216647	C3orf38	A/G	-0.027	0.003	1.79×10^{-16}	-0.052	0.023	0.026
rs1535793	13:47154966	LRCH1	G/A	-0.022	0.003	2.07×10^{-16}	-0.001	0.018	0.960
rs13073970	3:170630520	EIF5A2	T/G	0.024	0.003	2.73×10^{-16}	-0.001	0.02	0.968
rs10860878	12:102963550	IGF1	T/C	-0.020	0.002	2.92×10^{-16}	0.004	0.025	0.867
rs34536443	19:10463118	TYK2	C/G	-0.047	0.006	3.11×10^{-16}	-0.265	0.052	0.000
rs11568828	17:61996273	GH1	C/T	-0.032	0.004	4.82×10^{-16}	-0.042	0.064	0.513
rs6853741	4:148982559	ARHGAP10	G/A	-0.022	0.003	5.58×10^{-16}	0.018	0.019	0.329
rs78511209	1:91414243	ZNF644	G/C	0.033	0.004	7.88×10^{-16}	-0.037	0.032	0.257
rs273951	7:137605504	CREB3L2	T/A	0.019	0.002	9.47×10^{-16}	0.004	0.017	0.800
rs75660441	9:97662448	C9orf3	G/A	-0.038	0.005	2.26×10^{-15}	0.030	0.034	0.381
rs144588154	4:90004986	FAM13A	TTTA/T	0.024	0.003	3.31×10^{-15}	-0.005	0.021	0.818
rs12491473	3:46989904	CCDC12	A/G	-0.019	0.002	3.31×10^{-15}	-0.063	0.017	0.000
rs1291208	20:62330417	ARFRP1	T/C	0.034	0.004	3.36×10^{-15}	-0.143	0.032	0.000
rs117120687	15:68342718	PIAS1	T/A	-0.044	0.006	3.49×10^{-15}	-0.032	0.045	0.477
rs113439442	16:1995997	RPL3L	T/C	-0.039	0.005	5.05×10^{-15}	-0.079	0.062	0.197
rs10142298	14:93909792	UNC79	C/T	-0.020	0.003	6.12×10^{-15}	-0.012	0.017	0.503
rs296361	19:48389363	SULT2A1	A/G	0.025	0.003	7.75×10^{-15}	0.004	0.022	0.868
rs3858325	10:117988795	GFRA1	T/C	0.018	0.002	7.97×10^{-15}	0.009	0.016	0.580
rs5755943	22:36166493	RBFOX2	C/G	0.027	0.003	8.49×10^{-15}	-0.044	0.025	0.079
rs1260003	12:32086561	KIAA1551	C/T	0.019	0.003	8.79×10^{-15}	0.010	0.017	0.553
rs56992534	3:98568732	DCBLD2	T/TA	0.019	0.002	8.96×10^{-15}	-0.017	0.016	0.306
rs998584	6:43757896	VEGFA	A/C	0.018	0.002	1.10×10^{-14}	-0.021	0.018	0.237
rs10757291	9:22161884	CDKN2B	G/A	0.018	0.002	1.17×10^{-14}	-0.009	0.016	0.566

rs798545	7:2762386	AMZ1	T/C	0.020	0.003	1.67×10^{-14}	0.009	0.018	0.603
rs12179053	6:160711566	SLC22A2	T/C	-0.021	0.003	1.79×10^{-14}	-0.004	0.019	0.829
rs7719168	5:53292390	ARL15	C/A	0.029	0.004	1.85×10^{-14}	0.029	0.026	0.254
rs1431015	8:77131580	ZFHX4	T/C	-0.018	0.002	1.95×10^{-14}	-0.004	0.017	0.797
rs111516782	4:121723035	PRDM5	TATATA/T	0.020	0.003	2.24×10^{-14}	-0.037	0.018	0.033
rs61115258	19:30718774	ZNF536	G/C	-0.021	0.003	2.34×10^{-14}	0.025	0.019	0.191
rs62263343	3:107239783	BBX	A/G	-0.024	0.003	3.09×10^{-14}	-0.002	0.021	0.914
rs727428	17:7537792	SHBG	T/C	-0.018	0.002	3.39×10^{-14}	0.024	0.018	0.175
rs9978775	21:40694526	BRWD1	A/G	-0.018	0.002	3.89×10^{-14}	-0.003	0.016	0.870
rs4822452	22:24254363	MIF	C/T	0.018	0.002	4.10×10^{-14}	0.014	0.017	0.395
rs1046011	1:65898996	LEPR	T/C	0.019	0.003	4.73×10^{-14}	-0.027	0.019	0.151
rs117104648	11:65543736	AP5B1	C/T	0.037	0.005	4.78×10^{-14}	0.233	0.058	0.000
rs11928797	3:33457493	UBP1	A/C	0.028	0.004	5.47×10^{-14}	0.047	0.028	0.095
rs11763787	7:31019597	GHRHR	A/G	-0.019	0.003	7.22×10^{-14}	-0.013	0.018	0.466
rs11423985	6:147601203	STXBP5	CT/C	-0.018	0.002	7.73×10^{-14}	0.020	0.016	0.230
rs8095538	18:1616505	ADCYAP1	G/T	0.019	0.003	8.08×10^{-14}	-0.018	0.018	0.306
rs329123	5:133865435	JADE2	T/A	0.019	0.003	1.47×10^{-13}	0.050	0.018	0.005
rs7012213	8:135660469	ZFAT	T/A	0.019	0.003	1.76×10^{-13}	0.001	0.017	0.964
rs36086195	1:16510894	ARHGEF19	C/T	0.018	0.002	1.86×10^{-13}	-0.017	0.017	0.315
rs11029620	11:3771924	NUP98	T/C	-0.021	0.003	2.30×10^{-13}	0.020	0.02	0.305
rs6933547	6:37969064	ZFAND3	C/A	-0.019	0.003	2.35×10^{-13}	0.020	0.018	0.269
rs10828250	10:21847178	MLLT10	G/C	-0.019	0.003	2.58×10^{-13}	0.037	0.018	0.037
rs55686478	8:18250375	NAT2	A/G	0.024	0.003	2.63×10^{-13}	0.016	0.023	0.479
rs2724373	1:207999200	CD46	T/C	-0.018	0.002	2.69×10^{-13}	-0.044	0.017	0.011
rs6602909	13:114551993	GAS6	C/T	0.018	0.003	3.21×10^{-13}	0.004	0.018	0.809
rs2123947	1:91598985	ZNF644	G/C	0.017	0.002	3.60×10^{-13}	-0.013	0.016	0.438
rs2450137	11:77925233	GAB2	G/C	-0.023	0.003	4.27×10^{-13}	0.041	0.022	0.060
rs76750172	13:28395297	GSX1	T/C	0.056	0.008	4.27×10^{-13}	0.097	0.072	0.179

rs11620783	14:24871530	NYNRIN	T/C	-0.017	0.002	5.18×10^{-13}	0.033	0.018	0.070
rs2208030	20:3355567	C20orf194	T/C	0.017	0.002	5.70×10^{-13}	0.036	0.016	0.027
rs657536	6:100086484	PRDM13	G/C	-0.018	0.003	7.17×10^{-13}	0.019	0.018	0.288
rs7545345	1:205690941	NUCKS1	C/T	0.025	0.004	7.83×10^{-13}	0.007	0.024	0.779
rs175043	14:75471803	EIF2B2	A/G	-0.017	0.002	9.89×10^{-13}	0.023	0.016	0.152
rs60933741	12:116196548	MED13L	C/T	0.031	0.004	1.06×10^{-12}	0.050	0.041	0.225
rs12454712	18:60845884	BCL2	C/T	-0.017	0.002	1.08×10^{-12}	0.001	0.017	0.972
rs12666306	7:115082406	MDFIC	G/A	0.017	0.002	1.18×10^{-12}	0.010	0.016	0.558
rs61866607	10:95323258	FFAR4	T/A	0.040	0.006	1.29×10^{-12}	0.055	0.038	0.151
rs6473015	8:78178485	PEX2	C/A	0.018	0.003	1.78×10^{-12}	-0.002	0.018	0.917
rs11181153	12:38289817	ALG10B	C/T	0.017	0.002	1.88×10^{-12}	-0.017	0.018	0.341
rs2207132	20:39142516	MAFB	A/G	-0.046	0.007	1.89×10^{-12}	0.052	0.211	0.807
rs3762311	1:65355938	JAK1	G/T	0.026	0.004	2.00×10^{-12}	-0.084	0.027	0.002
rs17714046	5:180661980	TRIM41	C/T	0.039	0.006	2.07×10^{-12}	0.142	0.072	0.050
rs62560860	9:34077462	DCAF12	A/G	0.021	0.003	2.22×10^{-12}	0.041	0.02	0.044
rs75120545	2:44271496	LRPPRC	T/C	0.051	0.007	2.27×10^{-12}	0.041	0.058	0.480
rs7589901	2:113993344	PAX8	C/A	0.017	0.003	2.39×10^{-12}	-0.015	0.017	0.386
rs12535428	7:113858363	FOXP2	T/C	0.018	0.003	2.55×10^{-12}	0.015	0.018	0.391
rs1465529	2:231039037	SP110	C/T	-0.018	0.003	2.64×10^{-12}	0.001	0.018	0.946
rs11603111	11:1568192	DUSP8	C/A	0.017	0.002	2.81×10^{-12}	-0.032	0.017	0.053
rs61780439	1:41490177	SCMH1	A/G	-0.020	0.003	2.84×10^{-12}	0.011	0.02	0.558
rs59929354	16:1005057	LMF1	C/T	0.017	0.002	3.03×10^{-12}	-0.014	0.017	0.396
rs10821713	10:62055781	ANK3	T/C	0.017	0.002	3.23×10^{-12}	-0.009	0.017	0.581
rs114949263	7:150498245	TMEM176B	C/T	-0.026	0.004	4.57×10^{-12}	-0.005	0.027	0.858
rs10777540	12:94150321	CRADD	T/G	-0.016	0.002	4.65×10^{-12}	-0.023	0.016	0.163
rs1562617	2:6384728	SOX11	A/G	-0.020	0.003	4.73×10^{-12}	0.010	0.02	0.592
rs1112847	6:130335109	L3MBTL3	C/T	0.016	0.002	5.98×10^{-12}	-0.014	0.016	0.390
rs1786342	8:101676363	SNX31	C/T	-0.017	0.002	6.43×10^{-12}	-0.004	0.017	0.814

rs116509476	1:220693476	MARK1	C/T	0.050	0.007	6.56×10^{-12}	0.091	0.054	0.091
rs17037452	1:11895675	CLCN6	G/A	-0.022	0.003	7.37×10^{-12}	0.017	0.022	0.450
rs10869022	9:74057313	TMEM2	T/C	-0.020	0.003	8.28×10^{-12}	-0.005	0.021	0.804
rs10498238	2:230166564	PID1	T/C	0.030	0.005	8.39×10^{-12}	-0.048	0.031	0.118
rs7910087	10:77209145	C10orf11	T/C	0.016	0.002	9.55×10^{-12}	-0.024	0.017	0.151
rs10892564	11:120224650	ARHGEF12	G/A	0.016	0.002	1.03×10^{-11}	-0.007	0.017	0.662
rs4547160	12:63503650	AVPR1A	T/G	0.017	0.003	1.26×10^{-11}	-0.021	0.018	0.235
rs9896243	17:44826056	NSF	G/C	0.019	0.003	1.74×10^{-11}	-0.053	0.02	0.010
rs12051698	17:79046374	BAIAP2	G/C	-0.017	0.003	1.75×10^{-11}	0.007	0.018	0.684
rs12244388	10:104640052	AS3MT	A/G	-0.017	0.003	1.89×10^{-11}	-0.002	0.017	0.906
rs6437249	2:242175331	HDLBP	T/C	-0.017	0.003	2.06×10^{-11}	0.014	0.018	0.436
rs34669210	11:122772285	C11orf63	T/TA	-0.016	0.002	2.18×10^{-11}	-0.017	0.017	0.317
rs2607748	3:14158725	CHCHD4	C/T	0.016	0.002	2.42×10^{-11}	-0.008	0.017	0.638
rs9879333	3:186381220	HRG	A/G	-0.018	0.003	2.69×10^{-11}	-0.022	0.019	0.247
rs10798673	1:179342753	AXDND1	T/C	0.019	0.003	2.96×10^{-11}	0.010	0.021	0.626
rs112941090	16:69130106	HAS3	G/C	-0.021	0.003	4.15×10^{-11}	0.003	0.022	0.892
rs6974707	7:55982894	ZNF713	A/G	0.019	0.003	4.19×10^{-11}	0.022	0.02	0.261
rs26822	5:102518795	PPIP5K2	G/A	0.017	0.003	4.21×10^{-11}	0.003	0.018	0.869
rs12935465	16:17476853	XYLT1	C/T	-0.016	0.002	4.41×10^{-11}	-0.016	0.016	0.328
rs78607331	12:57648644	R3HDM2	T/C	-0.037	0.006	4.68×10^{-11}	-0.032	0.064	0.619
rs2762981	13:21487753	XPO4	G/A	-0.018	0.003	5.97×10^{-11}	-0.022	0.021	0.279
rs1170187	13:42679835	DGKH	G/A	-0.020	0.003	6.63×10^{-11}	0.023	0.021	0.272
rs118018874	16:1969877	HS3ST6	T/C	-0.067	0.01	7.47×10^{-11}	-0.066	0.138	0.632
rs9292578	5:35230075	PRLR	A/C	-0.038	0.006	7.50×10^{-11}	0.003	0.042	0.937
rs72858776	11:15772953	SOX6	T/G	-0.028	0.004	7.63×10^{-11}	0.039	0.033	0.238
rs6913063	6:129326573	LAMA2	C/G	0.016	0.003	7.86×10^{-11}	0.031	0.018	0.083
rs1430753	1:68692642	WLS	A/G	0.020	0.003	8.32×10^{-11}	0.004	0.021	0.856
rs7594734	2:42705282	KCNG3	C/G	-0.023	0.004	9.93×10^{-11}	-0.013	0.025	0.592

rs35887778	7:99831168	GATS	C/T	0.018	0.003	1.00×10^{-10}	0.007	0.021	0.749
rs17699089	19:11343795	DOCK6	G/A	0.025	0.004	1.00×10^{-10}	-0.008	0.027	0.750
rs58912472	17:1626018	WDR81	A/G	0.015	0.002	1.04×10^{-10}	-0.037	0.017	0.027
rs2366398	5:89437963	CETN3	T/G	0.018	0.003	1.19×10^{-10}	0.011	0.02	0.585
rs112893170	3:57211863	IL17RD	C/T	-0.019	0.003	1.23×10^{-10}	0.046	0.022	0.037
rs68115887	7:138200210	TRIM24	C/T	0.016	0.003	1.45×10^{-10}	-0.007	0.017	0.689
rs4720478	7:44784259	ZMIZ2	C/A	0.015	0.002	1.54×10^{-10}	-0.021	0.017	0.218
rs6486121	11:13355770	ARNTL	C/T	-0.016	0.002	1.54×10^{-10}	-0.001	0.016	0.941
rs13108218	4:3443931	HGFAC	A/G	-0.016	0.002	1.60×10^{-10}	0.018	0.018	0.336
rs11031002	11:30215261	FSHB	A/T	0.022	0.004	1.70×10^{-10}	-0.032	0.024	0.191
rs8084239	18:50725943	DCC	T/C	0.015	0.002	1.75×10^{-10}	0.018	0.016	0.275
rs55843942	1:214219331	PROX1	G/C	-0.022	0.004	1.87×10^{-10}	0.124	0.043	0.004
rs117292219	17:40451137	STAT5A	T/G	-0.026	0.004	2.19×10^{-10}	0.045	0.034	0.184
rs4823324	22:46238123	ATXN10	C/T	-0.015	0.002	2.23×10^{-10}	-0.003	0.016	0.849
rs61902812	11:113374420	DRD2	A/C	0.015	0.002	2.58×10^{-10}	0.002	0.017	0.904
rs4709989	6:166260182	PDE10A	A/G	-0.017	0.003	2.84×10^{-10}	0.001	0.02	0.959
rs7228277	18:75150503	GALR1	A/G	-0.015	0.002	2.95×10^{-10}	-0.026	0.017	0.117
rs903908	1:2202967	SKI	T/C	-0.015	0.002	3.07×10^{-10}	0.005	0.017	0.770
rs4678732	3:33212485	SUSD5	A/G	0.015	0.002	3.32×10^{-10}	-0.004	0.017	0.811
rs2091503	2:73687865	ALMS1	A/G	-0.015	0.002	3.92×10^{-10}	-0.005	0.017	0.762
rs33384	5:142609525	ARHGAP26	C/G	-0.017	0.003	3.92×10^{-10}	-0.083	0.018	0.000
rs7574340	2:40621239	SLC8A1	T/C	0.016	0.003	4.30×10^{-10}	0.030	0.018	0.100
rs112481283	15:40931357	KNL1	CT/C	0.015	0.002	4.79×10^{-10}	-0.014	0.016	0.381
rs8055075	16:56124884	GNAO1	C/G	0.015	0.002	5.03×10^{-10}	0.024	0.016	0.149
rs9657541	8:10643164	PINX1	T/C	-0.018	0.003	5.03×10^{-10}	-0.022	0.021	0.284
rs533123	1:29141155	OPRD1	G/A	0.018	0.003	5.04×10^{-10}	-0.008	0.021	0.719
rs13418037	2:218314141	TNS1	T/C	0.019	0.003	5.14×10^{-10}	-0.010	0.021	0.639
rs6462426	7:32905809	KBTBD2	T/C	0.032	0.005	5.86×10^{-10}	0.026	0.035	0.449

rs1351893	5:173374754	CPEB4	A/T	0.028	0.005	6.51×10^{-10}	0.005	0.034	0.872
rs9583151	13:107666257	FAM155A	C/T	0.015	0.002	6.62×10^{-10}	0.047	0.016	0.004
rs939626	15:99493176	IGF1R	C/T	0.015	0.002	6.67×10^{-10}	-0.006	0.016	0.712
rs17258904	6:21928131	SOX4	G/A	0.016	0.003	6.70×10^{-10}	0.005	0.018	0.792
rs282177	1:26899125	RPS6KA1	T/C	0.017	0.003	6.74×10^{-10}	0.049	0.018	0.009
rs4296683	4:97570279	PDHA2	T/C	-0.015	0.002	7.26×10^{-10}	-0.011	0.017	0.514
rs116971887	16:51170026	SALL1	T/G	-0.035	0.006	7.85×10^{-10}	0.010	0.042	0.816
rs62334147	4:169345005	DDX60L	C/T	0.019	0.003	8.37×10^{-10}	0.008	0.021	0.708
rs7114896	11:2059546	MRPL23	A/C	-0.014	0.002	9.58×10^{-10}	0.015	0.017	0.372
rs2925656	8:59237034	UBXN2B	A/C	0.017	0.003	9.97×10^{-10}	-0.021	0.02	0.295
rs6496499	15:89111922	DET1	G/A	0.015	0.002	1.15×10^{-9}	-0.006	0.017	0.727
rs518640	11:94232883	ANKRD49	C/T	0.015	0.003	1.18×10^{-9}	-0.006	0.017	0.725
rs2268829	3:185989567	DGKG	G/A	0.017	0.003	1.23×10^{-9}	-0.019	0.02	0.343
rs1150781	6:34214322	C6orf1	C/G	-0.025	0.004	1.26×10^{-9}	0.046	0.037	0.219
rs4789227	17:73794354	UNK	C/T	-0.015	0.003	1.39×10^{-9}	0.056	0.017	0.001
rs7146217	14:69250915	ZFP36L1	T/C	0.014	0.002	1.42×10^{-9}	0.087	0.016	0.000
rs7817124	8:81404008	ZBTB10	C/G	-0.017	0.003	1.45×10^{-9}	0.025	0.019	0.188
rs2801482	10:12459773	CAMK1D	G/A	0.047	0.008	1.47×10^{-9}	-0.054	0.049	0.262
rs2309401	17:5471902	NLRP1	G/T	-0.015	0.002	1.54×10^{-9}	-0.010	0.017	0.569
rs9379083	6:7221443	RREB1	A/G	0.017	0.003	2.08×10^{-9}	0.000	0.035	0.996
rs585187	18:58177124	MC4R	T/G	0.014	0.002	2.43×10^{-9}	0.011	0.016	0.518
rs2846579	18:901897	ADCYAP1	T/C	0.020	0.003	2.44×10^{-9}	-0.015	0.025	0.545
rs376096585	2:56094578	EFEMP1	CT/C	0.017	0.003	2.61×10^{-9}	-0.035	0.019	0.063
rs58368073	16:19275918	SYT17	CTTT/C	-0.014	0.002	2.67×10^{-9}	-0.036	0.017	0.031
rs4501681	9:19440040	ACER2	C/T	0.020	0.003	2.83×10^{-9}	-0.009	0.022	0.693
rs7899156	10:22822524	PIP4K2A	C/A	-0.015	0.003	2.91×10^{-9}	-0.001	0.016	0.956
rs3891689	9:119258583	ASTN2	C/T	0.016	0.003	3.15×10^{-9}	0.004	0.02	0.843
rs2812536	10:71319402	NEUROG3	G/C	-0.015	0.003	3.21×10^{-9}	-0.027	0.019	0.157

rs232000	3:172270021	TNFSF10	A/G	0.015	0.003	3.47×10^{-9}	-0.009	0.018	0.613
rs35628191	2:27004254	SLC35F6	T/C	0.014	0.002	3.56×10^{-9}	0.010	0.017	0.564
rs11064536	12:905582	WNK1	C/T	-0.018	0.003	3.57×10^{-9}	-0.018	0.023	0.420
rs76979176	3:51264296	DOCK3	A/G	0.046	0.008	3.67×10^{-9}	-0.272	0.168	0.106
rs7323205	13:110365525	IRS2	T/C	-0.014	0.002	3.68×10^{-9}	0.000	0.016	0.985
rs80170948	5:64020316	SREK1IP1	G/T	0.035	0.006	4.15×10^{-9}	0.079	0.107	0.459
rs293275	10:53215020	PRKG1	T/C	-0.014	0.002	4.59×10^{-9}	0.018	0.016	0.266
rs411717	7:94033031	COL1A2	T/C	0.014	0.002	4.69×10^{-9}	0.004	0.017	0.790
rs347617	3:11278211	HRH1	C/T	-0.015	0.003	4.76×10^{-9}	-0.003	0.017	0.865
rs3770183	2:222313445	EPHA4	A/T	-0.014	0.002	4.80×10^{-9}	-0.017	0.016	0.287
rs7658049	4:129158203	LARP1B	T/G	-0.017	0.003	5.00×10^{-9}	0.004	0.019	0.845
rs28396553	14:36673392	MBIP	C/T	-0.014	0.002	5.38×10^{-9}	0.037	0.018	0.044
rs74218214	10:39149441	ZNF37A	T/C	0.016	0.003	5.66×10^{-9}	0.081	0.052	0.116
rs2802951	1:235014058	TOMM20	A/G	0.015	0.003	5.82×10^{-9}	-0.009	0.018	0.632
rs13069961	3:124358715	KALRN	G/A	0.017	0.003	6.21×10^{-9}	0.030	0.02	0.134
rs58387407	2:152924773	CACNB4	G/A	0.017	0.003	6.78×10^{-9}	0.003	0.02	0.887
rs36010444	20:20374002	RALGAPA2	A/G	-0.015	0.003	6.99×10^{-9}	-0.012	0.018	0.499
rs12712929	2:45192105	SIX3	T/G	-0.018	0.003	7.90×10^{-9}	-0.038	0.025	0.132
rs2298604	8:109271776	EIF3E	G/A	-0.016	0.003	7.95×10^{-9}	0.004	0.02	0.839
rs3809627	16:30103160	TBX6	A/C	-0.014	0.002	8.14×10^{-9}	-0.097	0.018	0.000
rs77704058	2:111928231	BCL2L11	G/C	0.030	0.005	8.24×10^{-9}	0.050	0.038	0.185
rs55848514	5:142902056	NR3C1	T/C	0.015	0.003	8.36×10^{-9}	-0.020	0.018	0.267
rs10835211	11:27701365	BDNF	A/G	-0.015	0.003	8.41×10^{-9}	0.042	0.019	0.024
rs7952602	11:126233669	ST3GAL4	C/G	-0.020	0.004	8.66×10^{-9}	-0.068	0.025	0.008
rs12978130	19:4053428	ZBTB7A	C/T	-0.015	0.003	8.94×10^{-9}	-0.044	0.02	0.032
rs8079923	17:19869544	AKAP10	T/C	-0.015	0.003	9.53×10^{-9}	0.008	0.019	0.651
rs1498603	5:58333125	PDE4D	G/T	-0.027	0.005	9.95×10^{-9}	0.100	0.035	0.004
rs10876864	12:56401085	SUOX	G/A	-0.014	0.002	1.02×10^{-8}	-0.011	0.017	0.489

rs2997468	10:81073763	ZMIZ1	C/G	0.014	0.003	1.47×10^{-8}	-0.030	0.019	0.114
rs12609574	19:7178517	INSR	T/G	-0.014	0.003	1.51×10^{-8}	0.026	0.018	0.132
rs4890489	18:42431244	SETBP1	A/G	0.013	0.002	1.57×10^{-8}	-0.022	0.016	0.191
rs1260631	19:34720258	LSM14A	C/A	-0.014	0.002	1.59×10^{-8}	-0.008	0.017	0.628
rs17523284	15:51538723	CYP19A1	A/G	-0.013	0.002	1.73×10^{-8}	0.028	0.016	0.082
rs5784791	10:50317533	VSTM4	AT/A	0.014	0.003	1.76×10^{-8}	-0.005	0.017	0.752
rs7457999	7:133748235	EXOC4	C/T	0.015	0.003	1.80×10^{-8}	0.012	0.021	0.569
rs12108803	5:77158507	TBCA	G/T	0.030	0.006	1.84×10^{-8}	0.030	0.04	0.458
rs12520308	5:111253269	NREP	T/C	-0.014	0.003	1.84×10^{-8}	0.005	0.017	0.767
rs2296198	6:18399750	RNF144B	T/C	-0.015	0.003	2.01×10^{-8}	0.026	0.019	0.166
rs2723833	12:12055435	ETV6	C/G	0.013	0.002	2.04×10^{-8}	-0.068	0.017	0.000
rs62206964	20:62185011	FNDC11	C/T	-0.014	0.002	2.09×10^{-8}	0.050	0.038	0.191
rs2875238	11:130282078	ADAMTS8	T/C	0.014	0.003	2.14×10^{-8}	-0.011	0.018	0.556
rs10246481	7:156184748	RNF32	G/A	0.013	0.002	2.26×10^{-8}	-0.004	0.017	0.794
rs11788077	9:86700760	RMI1	G/A	-0.014	0.003	2.41×10^{-8}	0.014	0.02	0.492

Note: IGF-1, insulin-like growth factor-1; MS, multiple sclerosis; SNP, Single-nucleotide polymorphism; EA, effect allele; OA, other allele.

Supplementary Table 5. Summary statistics utilized in the Mendelian randomization study of IGFBP-3 on MS

SNP	Coordinate (GRCh37)	Gene	EA/OA	Association with IGFBP-3			Association with MS		
				Beta	Se	*P*-value	Beta	Se	*P*-value
rs11977526	7:46008110	IGFBP3	A/G	0.287	0.011	2.31×10^{-150}	0.018	0.017	0.294
rs700753	7:46753684	TNS3	C/G	-0.158	0.011	4.38×10^{-47}	0.005	0.017	0.774
rs1065656	16:1838836	NUBP2	C/G	-0.111	0.011	3.03×10^{-24}	0.003	0.018	0.871
rs4234798	4:7219933	SORCS2	T/G	-0.095	0.011	2.90×10^{-18}	0.023	0.017	0.170

Note: IGFBP-3, insulin-like growth factor-binding protein 3; MS, multiple sclerosis.

Supplementary Table 6. Summary statistics utilized in the Mendelian randomization study of VEGF on MS

SNP	Coordinate (GRCh37)	Gene	EA/OA	Association with VEGF			Association with MS		
				Beta	Se	*P*-value	Beta	Se	*P*-value
rs6921438	6:43925607	LOC100132[illegible]	A/G	-0.64	0.008	1.00×10^{-200}	0.014	0.017	0.418
rs2375981	9:2692583	KCNV2	G/C	-0.21	0.01	9.49×10^{-99}	0.000	0.022	0.993
rs6993770	8:106581528	ZFPM2	T/A	-0.16	0.01	3.83×10^{-55}	0.01	0.019	0.592
rs10761741	10:65066186	JMJD1C	G/T	-0.08	0.009	2.99×10^{-19}	0.001	0.017	0.948
rs11965885	6:43693094	VEGFA	T/G	-0.09	0.01	1.83×10^{-17}	-0.038	0.017	0.028
rs1740073	6:43947398	C6orf223	C/T	-0.09	0.01	4.40×10^{-17}	-0.029	0.017	0.100
rs7043199	9:2621145	VLDLR-AS[illegible]	A/T	-0.1	0.013	4.16×10^{-14}	-0.024	0.023	0.291
rs114694170	5:88180196	MEF2C	T/C	-0.15	0.023	1.09×10^{-11}	0.082	0.051	0.109
rs2639990	18:72915551	ZADH2	C/T	-0.11	0.018	5.85×10^{-10}	-0.058	0.035	0.099
rs4782371	16:88568831	ZFPM1	T/G	-0.07	0.011	1.26×10^{-9}	-0.001	0.02	0.952

Note: VEGF, vascular endothelial growth factor; MS, multiple sclerosis; SNP, Single-nucleotide polymorphism; EA, effect allele; OA, other allele.

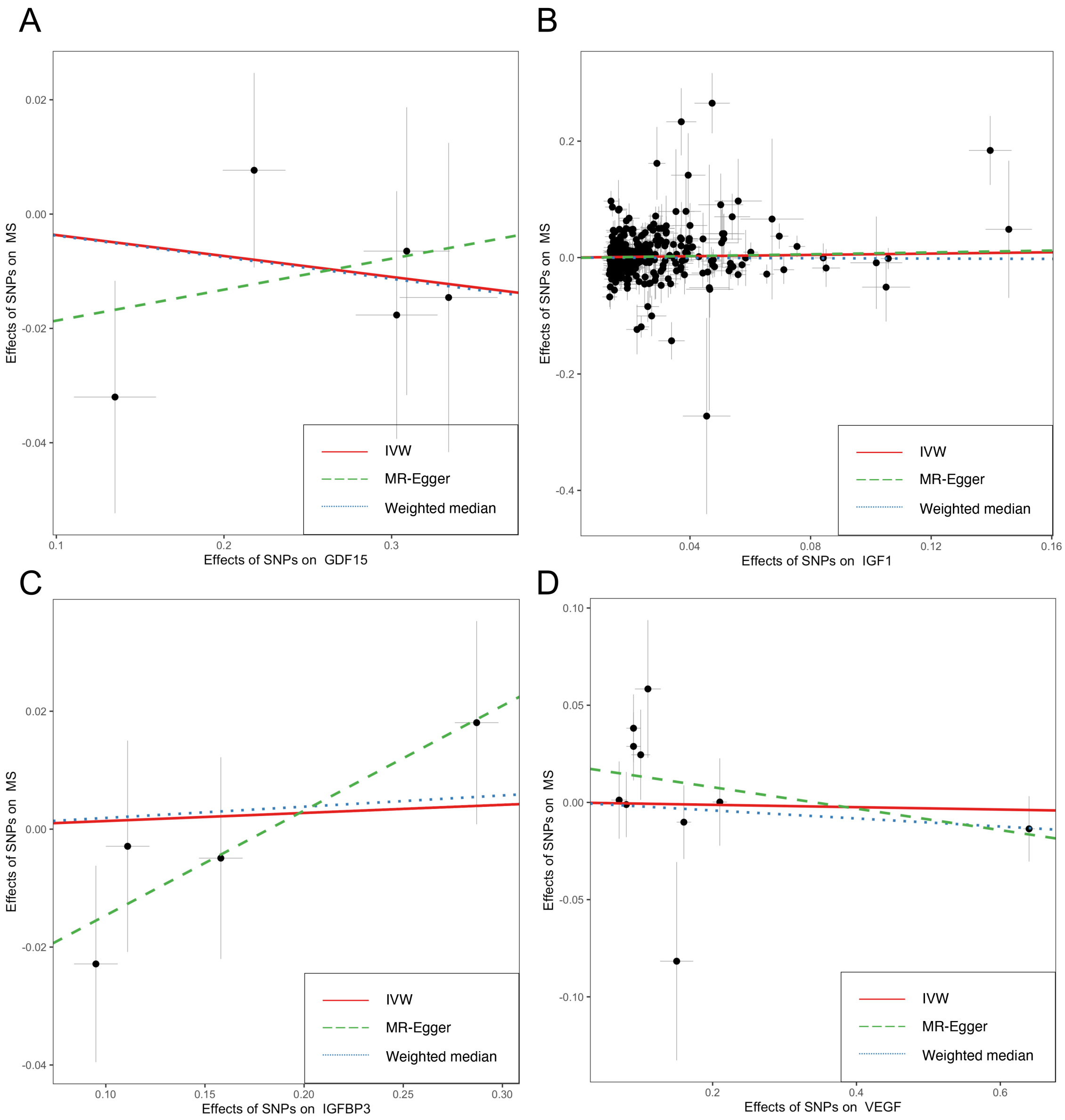

Supplementary Figure 1. Scatter plots in the Mendelian randomization analyses. GDF15, growth differentiation factor 15; IGF1, insulin-like growth factor 1; IGFBP3, insulin-like growth factor-binding protein 3; IVW, inverse variance weighted; SNP, Single-nucleotide polymorphism; VEGF, vascular endothelial growth factor; MS, multiple sclerosis.

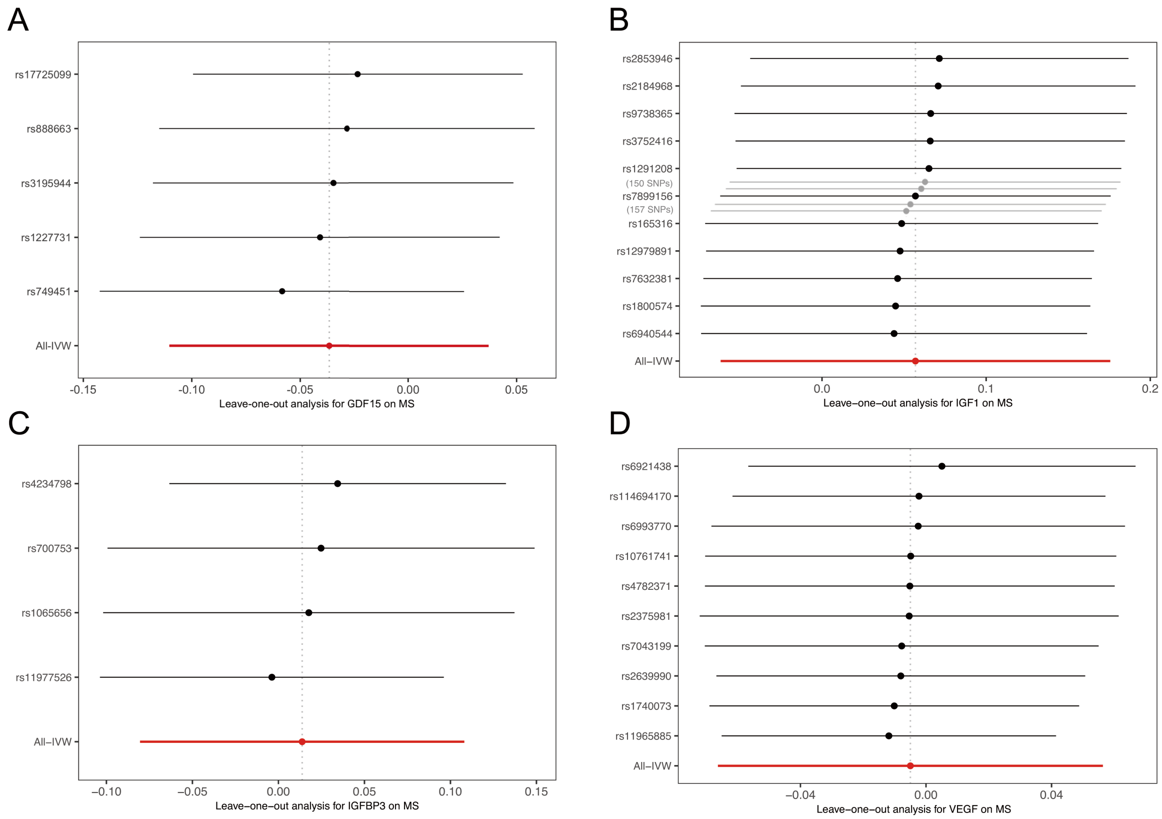

Supplementary Figure 2. Leave-one-out plots in the Mendelian randomization analyses. GDF15, growth differentiation factor 15; IGF1, insulin-like growth factor 1; IGFBP3, insulin-like growth factor-binding protein 3; IVW, inverse variance weighted; SNP, Single-nucleotide polymorphism; VEGF, vascular endothelial growth factor; MS, multiple sclerosis.
